# Risk of positive screening for anorexia nervosa, bulimia nervosa and night eating syndrome and associated risk factors in medical fellows in Northeastern Mexico: a multicenter study

**DOI:** 10.1186/s40337-023-00931-8

**Published:** 2023-12-07

**Authors:** Cynthia Isabel Ortiz-Lopez, Maria Elena Romero-Ibarguengoitia, Hector Cobos-Aguilar

**Affiliations:** 1 Departamento de Medicina Interna, Hospital Christus Muguerza Alta Especialidad, Miguel Hidalgo y Costilla 2525, Obispado, 64000 Monterrey, N.L. Mexico; 2 Especialidades Médicas, Escuela de Medicina, Universidad de Monterrey, Av. Ignacio Morones Prieto 4500-Pte, Zona Valle Poniente, 66238 San Pedro Garza García, N.L. Mexico; 3 Dirección de Enseñanza e Investigación en Salud, Hospital Christus Muguerza Sistemas de Salud, Pirineos 2707, Jardín, 64050 Monterrey, N.L. Mexico

**Keywords:** Eating disorders (EDs), Anorexia nervosa (AN), Bulimia nervosa (BN), Night eating syndrome (NES), Undergraduate medical interns (UMI), Medical residents (MR), Night eating syndrome questionnaire (NEQ)

## Abstract

**Background:**

Eating disorders and food ingestion (EDs) are serious mental illnesses with a higher prevalence in young adults, with difficult diagnoses that cause serious morbidity and mortality problems. There is not much information about the risk of positive screening for EDs, specifically, anorexia nervosa (AN) and bulimia nervosa (BN) and night eating syndrome (NES) in undergraduate medical interns (UMI) and medical residents (MR) in Mexico.

**Aim:**

To determine the risk of AN, BN and NES and to determine the risk factors of such conditions such as age, body mass index (BMI) and gender of MR and UMI with AN/BN and NES at four private hospitals in northeastern Mexico.

**Methods:**

A cross-sectional, descriptive, non-randomized survey in MR and UMI in four hospitals in Northeastern Mexico was conducted using an electronic questionnaire that included: informed consent signature, SCOFF questionnaire for AN and BN screening, NES questionnaire. Also, a survey on general sociodemographic data of each participant was included. Chi-square test and a logistic regression model were computed for analyses.

**Results:**

The population included a total of 129 MR and UMI. It was observed that 48.8% were positive for AN or BN and 32.6% were positive for the NES. There was no difference between age, sex, BMI, or medical specialty (if they were MR); however, MR from the first year had a higher risk of AN or BN (OR 23.7, 95% CI 1.181–475.266).

**Conclusions:**

There was a higher risk of positive screening for AN or BN and NES in UMI and MR in our population. In the case of MR, those in first year have a higher risk of AN and BN. Timely diagnosis and treatment are mandatory in this population.

## Abstract (plain language summary)

Eating disorders and food ingestion such as anorexia (AN) or bulimia (BN) nervosa and night eating syndrome (NES) are a group of mental illnesses that are frequently under diagnosed. Medical residents (MR) and undergraduate medical interns (UMI) are a high-risk population for such disorders due to their young age, stress environments, erratic eating patterns and long working hours. The aim of this study was to determine the risk of AN, BN and NES and to determine the risk factors of such conditions. One hundred twenty-nine UMI and MR were studied and showed that 48.8% were positive for AN or BN and 32.6% were positive for NES. MR in the first year of medical residency had a higher risk. Timely diagnosis and treatment are mandatory in this population.

## Background

Eating disorders and food ingestion (EDs) are serious mental illnesses that affects millions of individuals around the world, regardless of race, age, nationality, or sex, causing serious personal health, family, and social problems [1]. The importance of the diagnosis of EDs is due to the consequences they entail, such as increased comorbidities, high mortality, functional disability, and low recovery rates [2].

Among the different types of EDs included in the DSM-5 are anorexia nervosa (AN), bulimia nervosa (BN) and night eating syndrome (NES) [3]. AN entails restriction of food intake, intense fear of gaining weight and disturbance of body image; BN refers to the uncontrolled eating of a large amount of food in a short period followed by compensatory behaviours [4]. NES is characterized by morning anorexia, nocturnal hyperphagia, and insomnia and is associated with obesity and recurrent episodes of nocturnal eating [4].

Internationally, the prevalence of EDs in medical students is 10.4% according to systematic reviews and meta-analysis of literature, although it can vary from 2 to 30% in some studies [5]. Although it is difficult to establish the prevalence of EDs worldwide, it has been reported for the 2013–2018 period an estimate of 7.8% globally [6]. Specifically, the prevalence of BN is approximately four to six out of 200 women and for anorexia nervosa is one out of 200 women in the United States [4–6]. While the prevalence of NES is not well known globally; however, it is reported to be approximately 1.5% in the United States, being the main association with obesity; however, it has been inconsistent among the literature [7, 8]. In Mexico it is estimated that 10% of Mexican women have some degree of disordered eating behaviors, 1% of which will have severe consequences [9]. In Northeastern Mexico, there are few studies on EDs, even though the incidence of risky eating behaviors is 27.6%, exceeding the figure of 20% in the general population of our country [10].

In general, the risk factors associated with the development or maintenance of EDs are age, female gender, overweight or obesity. Also included are hereditary, cultural, social, social, geographic, psychological, environmental factors, and media influence [11–13].

Doctors in training are a high-risk group, since most of them are young people exposed to high levels of stress, long working hours, night shifts, few hours of sleep and erratic eating schedules, as well as a poor quality of food, all of which are risk factors for developing EDs [14–16]. There is not enough evidence of how prevalent anorexia, bulimia and NES are in undergraduate medical interns (UMI) and medical residents (MR) in Mexico and which risk factors are associated with. A study where the impact and risk factors can be determined is important because when EDs are correctly diagnosed and treated a complete recovery is possible [8, 17].

The aim of the study was to determine the risk for a positive screening for AN, BN and NES in UMI and MR and to determine if there are risk factors associated to these conditions.

## Methods

### Study design and population

This is a cross-sectional, descriptive, non-randomized survey based on an electronic, anonymous, self-administered survey in MR and UMI in four private hospitals in Northeastern Mexico. The aim of the study was to determine the risk for a positive screening for AN, BN and NES in UMI and MR and to determine if there are risk factors associated to these conditions such as age, gender, academic degree, medical specialty, personal medical history, and BMI with AN, BN and NES.

It was included by census sampling all MR and UMI who met the inclusion criteria: active enrollment in the hospitals included, signed informed consent, and completion of the questionnaire; and were excluded those who rejected to participate in our study from October 29, 2020, to August 30, 2022.

The survey was administered by means of a Google form via e-mail. It included four sections: informed consent, sociodemographic data (sex, age, academic grade, weight, height, MR or UMI; medical specialty, only appliable for MR, and personal medical history); for the screening of AN and BN the SCOFF questionnaire was used, it consists of 5 questions that require a yes/no answer; the questions are: do you make yourself vomit because you feel full, are you concerned that you have lost control over the amount of food you eat, have you lost weight in the last 3 month), do you think you are fat even though others say you are too thin, would you say that food dominates your life? A result of 2 positive answers would indicate the possibility of AN or BN [18, 19]. The SCOFF questionnaire developed by Morgan et al. has a sensitivity of 100% and specificity of 87.5% for diagnosis AN or BN separately and combined [20–22]. This questionnaire was validated in Mexican adults by Sánchez Armas [23]. NES was screened with Night Eating Questionnaire (NEQ) which is performed by a validated questionnaire containing 16 questions about the symptoms in the form of a scale from 0 to 4 on a Likert style scale that subsequently adds up the points, obtaining a positive result equal to or greater than 25. The criteria established by the 1st International Symposium on Nocturnal Ingestion in 2008, includes the presence of nocturnal hyperphagia, two or more nocturnal ingestions per week; and at least three of the following symptoms: morning anorexia, a strong urge to eat between dinner and sleep onset or during nighttime awakenings, insomnia at least four to five days a week, belief that eating is necessary to return to sleep, and depressed mood that worsens during nighttime hours. This questionnaire was validated by Allison et al. and translated to the Spanish version by Moizé et al. [24, 25].

### Statistical analysis

The sample size was all the MR and UMI who were actively enrolled, working shifts and studying, at the four private hospitals included in the study that accepted to participate and signed the inform consent. Descriptive statistics were performed for mean, standard deviation (normal distribution), frequencies, and percentages for demographic information of the population and prevalence of risk of screening positive for AN, BN and NES. The reliability of both questionnaires applied was determined with Cronbach's alpha. Chi-square test was performed to compare sex, academic degree, personal medical history, medical specialty, and type of specialty. Student's t-test was performed to compare BMI and age. Mann–Whitney U test for academic degree. To determine the risk factors associated to AN, BN and NES, a logistic regression model was performed for the dependent variables AN, BN, and NES comparing independent variables such as age, sex, year of medical specialty and medical specialty. A value of *p* ≤ 0.05 was considered statistically significant. Microsoft Excel spreadsheet and SPSS Statistics version 25 electronic program (IBM, New York, United States) were used to collect the database information.

## Results

The studied population included a total of 129 MR and UMI out of 241 doctors in training enrolled who answered the SCOFF and NES questionnaires (Table 1); with an overall response rate of 53.5%. Of the participants, 87 (67.4%) belonged to MR and 42 (32.5%) to UMI. Of all the MR, the first-year medical residents were the most to participate with a total of 40 (31%). According to medical specialty, the predominant specialty in terms of participation was internal medicine with 24 (18.6%). In line with the type of medical specialty (surgical or non-surgical), 30 MR (23.2%) were of surgical specialties and 57 (44.1%) in non-surgical specialties. According to gender, 69 (53.4%) women participated. The mean (SD) age was 26.5 years (2.49), and the mean BMI was 24 (4.28).

**Table 1:** Demographic and clinical characteristics

Population characteristics n = 129	Frequency (%)
Gender	
Woman	69 (53.4)
Age [mean (SD)] years	26.5 (2.49)
BMI kg/m^2^	
< 18.5	6 (4.6)
18.5–24.9	76 (58.9)
25–29.9	34 (26.3)
30–34.9	10 (7.7)
35–39.9	2 (1.5)
> 40	1 (0.7)
Academic degree	
UMI	42 (32.5)
MR	87 (67.4)
Personal medical history^a^	9 (6.9)
Specialty	
Internal medicine	24 (18.6)
General surgery	12 (9.3)
Gynecology	8 (6.2)
Pediatrics	10 (7.7)
Orthopedics	5 (3.8)
Anesthesiology	10 (7.7)
Quality in medical care	2 (1.5)
Pathology	3 (2.3)
Nuclear medicine	1 (0.7)
Radiology	8 (6.2)
Cardiology	2 (1.5)
Clinical pathology	2 (1.5)
Type of specialty	
Surgical	30 (23.2)
Non-surgical	57 (44.1)

^a^Personal medical history: diabetes mellitus, thyroid disease, cardiovascular disease, renal disease, others

*BMI* body mass index, *MR* medical resident, *UMI* undergraduate medical intern

We performed Cronbach’s alpha to determine the reliability of both questionnaires and we found it was 0.56 for the SCOFF questionnaire and 0.65 for the NEQ.

Regarding the results of the SCOFF and NEQ, 48.8% were positive for screening of AN, BN and 32.6% were positive for the screening of NES. The mean (SD) of SCOFF was 1.3 (1.09) and the mean (SD) of NEQ was 27.4 (8.6).

There was no difference for the screening of AN or BN between gender (*p* = 0.244), medical specialty (*p* = 0.616), personal medical history (*p* = 0.676) nor type of medical specialty (*p* = 0.531). In addition, there was no difference in age (*p* = 0.794) and BMI (*p* = 0.577). As well, no difference was found between gender (*p* = 0.581), age (*p* = 0.446), BMI (*p* = 0.306), medical specialty (*p* = 0.236), personal medical history (*p* = 0.430), and type of medical specialty (*p* = 0.348) for NES. Similarly, no difference was found for neither AN, BN (*p* = 0.639) and NES (*p* = 0.973) and year of specialty (Table 2).

**Table 2:** Comparison of participants characteristics with positive screening for AN, BN screening and NES

Population characteristics n = 129	Total frequency (%)	Positive screening for AN or BN frequency (%)	*p Value*	Positive for NES (%)	*p* Value
Gender					
Woman	69 (53.4)	37 (28.6)	0.244	21 (16.2)	0.581
Man	60 (46.5)	26 (20.1)		21 (16.2)	
Age [mean (SD)] years	26.5 (2.49)	–	0.794	–	0.446
BMI kg/m^2^					
< 18.5	6 (4.6)	–	0.577	–	0.306
18.5–24.9	76 (58.9)				
25–29.9	34 (26.3)				
30–34.9	10 (7.7)				
35–39.9	2 (1.5)				
> 40	1 (0.7)				
Academic degree					
UMI	42 (32.5)	22 (17)	0.616	15 (11.6)	0.236
MR	87 (67.4)	41 (31.7)		27 (20.9)	
Year of medical specialty					
1st year	40 (31)	–	0.639	–	0.973
2nd year	22 (17)				
3rd year	19 (14.7)				
4th year	6 (4.6)				
Personal medical history^a^	9 (6.9)	5 (3.8)	0.676	4 (3.1)	0.430
Specialty					
Internal medicine	24 (18.6)	13 (19.2)	0.616	4 (3.1)	0.236
General surgery	12 (9.3)	6 (9.6)		3 (2.3)	
Gynecology	8 (6.2)	3 (5.8)		2 (1.5)	
Pediatrics	10 (7.7)	4 (5.8)		4 (3.1)	
Orthopedics	5 (3.8)	4 (5.8)		4 (3.1	
Anesthesiology	10 (7.7)	6 (11.5)		3 (2.3)	
Quality in medical care	2 (1.5)	1 (1.9)		0	
Pathology	3 (2.3)	1 (1.9)		2 (1.5)	
Nuclear medicine	1 (0.7)	0		1 (0.7)	
Radiology	8 (6.2)	3 (3.8)		2 (1.5)	
Cardiology	2 (1.5)	0		1 (0.7)	
Clinical pathology	2 (1.5)	0		1 (0.7)	
Type of specialty					
Surgical	30 (23.2)	19 (14.7)	0.531	12 (9.3)	0.348
Non-surgical	57 (44.1)	22 (17)		15 (11.6)	

^a^Personal medical history: diabetes mellitus, thyroid disease, cardiovascular disease, renal disease, others. SCOFF questionnaire score ≥ 2 is considered positive in screening for AN and BN; NEQ score ≥ 25 is considered positive. This was performed using the chi-square statistical test for sex, academic degree, personal medical history, specialty and type of specialty, t-test was used for age and BMI and Mann–Whitney U test was used of year of specialty; *p* value < 0.05 was significant

A logistic regression model was performed for SCOFF questionnaire, and we found that MR in the first year of medical specialty had an OR of 23.7 of screening positive for AN or BN (*p* = 0.039). In addition, MR in their second year were almost at high risk for AN or BN (Table 3).Comparison group were subjects that had less than 2 yes answers. When regression was performed for NES, there were no risk factors.

**Table 3:** Comparison of participants characteristics with positive AN or BN nervosa screening

Population characteristics n = 129	B	SE	*p* value	Exp(B)	95% IC Lower	95% IC Upper
Gender	− 0.236	0.679	0.728	0.790	0.209	2.990
Age	0.476	0.280	0.089	1.610	0.930	2.788
Year of medical specialty						
1st year	3.165	1.530	0.039	23.694	1.181	475.266
2nd year	2.826	1.447	0.051	16.876	0.990	287.763
3rd year	1.407	1.283	0.273	4.082	0.330	50.441
Medical specialty	–	–	0.866	–	–	–

Binary logistic regression was performed for age, sex, medical specialty, and year of medical specialty. *p* Value < 0.05 was significant. Nagelkerke R square value of 0.3 * To consider SCOFF positive patients had to screen 2 or more yes answers. The reference group was subjects with less than 2 yes answers

## Discussion

The results of our study showed that there is a higher risk of a positive screening for AN or BN and NES in doctors in training in our population (48.8%) compared to the young population of northeastern Mexico (27.6%). The risk is also higher than the data previously reported in international studies on EDs in doctors in training (that varies according to studies between 10–30%) [11, 12].

### Risk of screening positive for AN, BN and NES

EDs are very frequently studied in the student population, generally in the adolescent population. There are few studies in graduate students and/or those studying a medical specialty or an undergraduate internship. Therefore, it is important to know the frequency of these disorders and to implement actions to improve their lifestyle.

In other Latin American countries such as Peru or Colombia, studies have been conducted where the prevalence of AN and BN in medical students has been reported to vary from 12.5 to 39.7% respectively, although it is important to emphasize that in both studies’ different questionnaires and instruments have been used for the detection of these disorders. Also, important to consider the cultural diversity of different countries and social customs [26–28].

On the other hand, the prevalence of NES in our population of 32.6% also exceeded that of the reviewed literature reported worldwide of 1.5%, although it is important to mention that there are not many studies on NES in the general population or in the medical student setting [8]. In a study conducted in Saudi Arabia in a medical student population, the prevalence was found to be 10% using the same instrument as our study. Although it is important to mention that in other studies that have been performed the cut-off point for considering positive NEQ varies from 25 or 30 [29].

### Risk factors associated with screening positively with AN, BN and NES

Although the prevalence was higher in our study population, no significant associations were found between the risk factors that have been described as the most common for developing EDs. There are associations that are already well documented, such as the risk of developing AN or BN with age, female sex, and an overweight or obese BMI for NES [12–16]. However there have been inconsistent association in the literature also [7, 8]. The most frequent risk factor in the literature is female sex, which in our study was not statistically significant [12]. However, we found that the first year of medical specialty were most likely to have a positive screening questionnaire for AN or BN. Our hypothesis is that the first year of a residency program is related to adjusting to a new environment with higher stress, a higher level of responsibility in the hospital with less expertise. Also, less time to eat and rest and high competitiveness and expectations of academic performance would make doctors in training start developing an eating disorder.

We also observed an increase in BMI in persons with at least one positive questionnaire, this may be due to an insufficient number of questionnaires completed, or to some other factor such as work stress or inadequate sleep quality, which were factors that were not considered in the study.

### Prevention and early detection of EDs

As mentioned above, EDs can have a variety of consequences; for example: risk of obesity, mental problems (depression, anxiety, among others), substance abuse, pharmacological measures to reduce weight, chronic stress that contributes to metabolic or cardiovascular diseases. Therefore, timely identification and prevention of these disorders is of utmost importance, although a main barrier to achieving this is the social stigma that exists around these disorders. It has been established that those who are exposed to prevention or early identification strategies increase their probability of recovery [30].

The strategies that currently exist for the prevention and management of EDs that have proven to be effective in reducing risk factors include identification of modifiable risk factors, promotion of healthy lifestyle, positive body image, balanced nutrition and physical activity, interactive tools that increase the participation of the young population and help them cope with the diagnosis and include long-term follow-up. However, there is currently insufficient information in the literature focused on EDs prevention programs that have a significant impact on reducing the frequency of EDs [31].

In the four private hospitals in our study, there are resources to support doctors in training by the affiliated university, such as the center for anxiety treatment and research, with which we could work to implement timely and early interventions on an individualized basis for fellow physicians who require both accompaniment and follow-up as well as channeling to more specialized help for their benefit. The inclusion of a multidisciplinary team providing support from a mental health professional has been reported in studies of EDs prevention as vital in prevention and early detection programs [32, 33].

### Limitations

It is also important to mention the limitations of the study, among which are the type of study, which was a cross-sectional survey and therefore no causal association was made. Also, the lack of parametric tests and inclusion of other sociodemographic variables that would be useful to extend the information on associated risk factors in our population, as well as the inclusion of another group of participants outside the medical area for comparative purposes. On the other hand, some of the data were provided by each participant subjectively and could be over or underestimated.

## Conclusions

There is a higher risk of screening positively for AN, BN and NES in our population of medical fellows compared to the general young population in Northeastern Mexico even though no associations were found with the most frequently described risk factors for such EDs; our study found significance in having a positive screening for AN or BN and being in first year of medical specialty.

It is of great importance to adequately screen and identify EDs in our population to provide the necessary support in case it is required and/or implement the necessary strategies.

Both questionnaires have the facility of being quick and self-applicable, so it is a simple form of screening to evaluate EDs and that may have a benefit in the population of fellow physicians.

## Data Availability

The database used and analyzed in this study is available from the corresponding author upon reasonable request.

## Abbreviations

EDs: Eating disorders
MR: Medical resident
UMI: Undergraduate medical intern
BMI: Body mass index
NES: Night eating syndrome
NEQ: Night eating syndrome questionnaire
SCOFF: Sick, control, one, fat, food
